# Insights into the Modulation of Dopamine Transporter Function by Amphetamine, Orphenadrine, and Cocaine Binding

**DOI:** 10.3389/fneur.2015.00134

**Published:** 2015-06-09

**Authors:** Mary Hongying Cheng, Ethan Block, Feizhuo Hu, Murat Can Cobanoglu, Alexander Sorkin, Ivet Bahar

**Affiliations:** ^1^Department of Computational and Systems Biology, School of Medicine, University of Pittsburgh, Pittsburgh, PA, USA; ^2^Department of Cell Biology, School of Medicine, University of Pittsburgh, Pittsburgh, PA, USA; ^3^Department of Pharmacology and Pharmaceutical Sciences, School of Medicine, Tsinghua University, Beijing, China

**Keywords:** human dopamine transporter, repurposable drugs, amphetamine, orphenadrine cocaine, drug modulation mechanism

## Abstract

Human dopamine (DA) transporter (hDAT) regulates dopaminergic signaling in the central nervous system by maintaining the synaptic concentration of DA at physiological levels, upon reuptake of DA into presynaptic terminals. DA translocation involves the co-transport of two sodium ions and the channeling of a chloride ion, and it is achieved via alternating access between outward-facing (OF) and inward-facing states of DAT. hDAT is a target for addictive drugs, such as cocaine, amphetamine (AMPH), and therapeutic antidepressants. Our recent quantitative systems pharmacology study suggested that orphenadrine (ORPH), an anticholinergic agent and anti-Parkinson drug, might be repurposable as a DAT drug. Previous studies have shown that DAT-substrates like AMPH or -blockers like cocaine modulate the function of DAT in different ways. However, the molecular mechanisms of modulation remained elusive due to the lack of structural data on DAT. The newly resolved DAT structure from *Drosophila melanogaster* opens the way to a deeper understanding of the mechanism and time evolution of DAT–drug/ligand interactions. Using a combination of homology modeling, docking analysis, molecular dynamics simulations, and molecular biology experiments, we performed a comparative study of the binding properties of DA, AMPH, ORPH, and cocaine and their modulation of hDAT function. Simulations demonstrate that binding DA or AMPH drives a structural transition toward a functional form predisposed to translocate the ligand. In contrast, ORPH appears to inhibit DAT function by arresting it in the OF open conformation. The analysis shows that cocaine and ORPH competitively bind DAT, with the binding pose and affinity dependent on the conformational state of DAT. Further assays show that the effect of ORPH on DAT uptake and endocytosis is comparable to that of cocaine.

## Introduction

The dopamine (DA) transporter (DAT) belongs to the SLC6 family of neurotransmitter:sodium symporters (NSSs, or solute carrier 6), structurally and functionally similar to transporters of serotonin (SERT) and norepinephrine (NET). DAT regulates the termination of dopaminergic signaling by reuptake of DA from synaptic clefts, assisted by the co-transport of Na^+^ ions down their electrochemical gradient and accompanied by Cl^-^ channeling. It is generally accepted that the NSS family functions through alternating access between outward-facing (OF) and inward-facing (IF) states (1). Uptake of substrate from the extracellular (EC) region takes place in the OF state and its release to the intracellular (IC) side, in the IF state. The OF and IF states may be open or closed depending on the local conformation of their respective EC- or IC-gating residues.

Dysfunction of hDAT has been implicated in many neurological diseases or psychiatric disorders [for reviews, see Ref. (2–4)], such as depression, Parkinson’s disease (PD) (5), epilepsy, autism (6), attention-deficit hyperactivity disorder (ADHD), obsessive–compulsive disorder, and Alzheimer’s disease. DAT is a target for addictive drugs and psychostimulants, such as cocaine and amphetamine (AMPH), and for therapeutic antidepressants. These modulate DAT structure and function through different mechanisms [review see Ref. (2)]. Cocaine acts as a DAT blocker by directly binding DAT and preventing the translocation of DA. AMPH, on the other hand, competes with DA and triggers the reverse transport (efflux) of DA from the cell interior to the synapse. Javitch, Galli, and Gnegy proposed that phosphorylation of one or more serines at the N-terminal end of DAT is essential for AMPH-induced DA efflux (7). AMPH also has indirect effects: it reverses the action of vesicular monoamine transporter 2 (VMAT2) (8) to stimulate the release of neurotransmitters (including serotonin, epinephrine in addition to DA) from synaptic vesicles to the presynaptic cell interior, and activates the trace amino-associated receptor 1 (TAAR1) (9) to trigger the efflux of DA by DAT. All these actions lead to increased synaptic DA levels, although the detailed mechanisms by which these effects take place remain unknown. Furthermore, these drugs may impact the trafficking of DATs [reviewed in Ref. (10)].

Human dopamine transporter has the same fold as leucine transporter (LeuT). LeuT has long served as a prototype for exploring NSS structure and function, being the first member of the NSS family, which has been structurally resolved at the atomic level (11). Despite its low (~20%) sequence identity to eukaryotic NSSs, the resolved LeuT structure (from the eubacteria homologue *Aqufex aeolicus*) provided valuable insights into the structural aspects of transport by eukaryotic NSS family members (12–20), mainly because its 3D architecture, *LeuT fold*, is shared by family members. The LeuT fold consists of 12 transmembrane (TM) helices organized in two pseudo-symmetric inverted repeats (11, 21): TM1–TM5 and TM6–TM10. TM1 and TM6 are broken near the substrate/ion-binding site. To date, LeuT has been crystallographically resolved in four conformations: substrate-bound OF *closed* (11) (shortly designated as OFc*, with the asterisk indicating substrate/ion-bound state), inhibitor-bound OF open (OFo*) (22), substrate-free OF *open* (OF*o*), and IF open (IF*o*) (23) conformations. Insights from structural data have been complemented by computational studies [e.g., molecular dynamics (MD) simulations] toward understanding the mechanism of transport by NSS family members (14, 16, 18–20, 24–32). In particular, the N-terminal segment of LeuT has been shown to play a key role in regulating the opening/closure of the IC gate and in resuming the transport cycle (24).

While structural models based on LeuT helped us make inferences on DAT structure and interactions, DAT differs from LeuT in terms of its sequence (sequence identity of 22%), detailed structure, as well as function. The recent resolution of the first eukaryotic DAT structure (33), dDAT, from *Drosophila melanogaster*, has opened the way to a structure-based exploration of DAT mechanism of function. dDAT has more than 50% sequence identity with hDAT (33). Moreover, the orientation of the TM12 helix in the dDAT crystal structure differs significantly from that in LeuT (33); and part of the DAT C-terminus is resolved for the first time. Although the dDAT structure lacks data on a 43-residue portion of the EC-exposed EL2 loop, which has been deleted (Δ164-206) in the crystal structure (33), it still serves as an excellent template for investigating the structure and dynamics of hDAT. The fact that the dDAT structure is resolved in the presence of nortriptyline (33), an antidepressant that inhibits transport, further helps in exploring the structural and dynamic bases of the actions of DAT substrates/blockers.

In addition to structure-based studies, there has been a surge in recent years in the number of quantitative systems pharmacology (QSP) approaches that exploit existing knowledge of protein–drug interactions. QSP approaches help reduce wet lab work, assist in selecting lead compounds, assessing side effects (34), and identifying repurposable drugs (35, 36). Most psychotropic drugs (e.g., clozapine, tricyclic antidepressants like amitriptyline) owe their efficacy to multiple interactions (37). QSP methods may thus be particularly useful in designing transporter blockers, which still is the most common strategy for antidepressant therapy in spite of known side effects (33, 38). We recently developed a probabilistic matrix factorization (PMF)-based method (39) that uses the known FDA-approved drug–target interactions as input to predict possible, but yet undisclosed interactions (40, 41).

In this study, we present an integrated computational and experimental study to elucidate the mechanism of interaction of drugs with DAT and their pharmacological implications. First, we combine the output from PMF computations with structural similarity analyses (42, 43), so as to extract potentially repurposable drugs (35, 36) for hDAT. The analysis highlights the potential significance of orphenadrine (ORPH), an inhibitor of NET reuptake (44), as a repurposable hDAT-inhibitor. A comparative MD study of ORPH, DA, and AMPH reveals the time-resolved mechanisms of binding and associated conformational changes; while DA uptake and endocytosis assays reveal the consequences on DAT function. Overall, our study suggests that ORPH inhibits DAT, like cocaine; while AMPH and DA share similar mechanisms of action and tend to stimulate the predisposition of DAT to substrate transport.

## Materials and Methods

### Computational material and methods

#### Combined PMF and 3D Structural Similarity Filtering of Known Drugs

Details of the PMF-based method for identifying repurposable drugs, side effects, and drug–drug or target–target similarities are described in our previous work and in the tutorial of the web interface that we developed to facilitate the use of the PMF-based package for QSP (39, 41). In a nutshell, the PMF uses as input a dataset of drug–target associations [e.g., those listed in DrugBank (45)]. The association profiles/patterns for each drug (or target) across all targets (or drugs) are used to make new inferences about potential drug–target interactions. In the present application, the repurposable drugs predicted by PMF for DAT as well as those known to bind/interact with DAT were clustered using agglomerative hierarchical cluster trees (Matlab). Three distance metrics (Euclidian, cosine and City-block/Mahalanobis) were used to extract the associations that were robustly predicted regardless of the metric. Dendogram enrichment technique was adopted to identify those clusters enriched in known drugs. The 3D structural similarities between drugs were examined using OpenEye^1^ toolkits OMEGA (42) and Shape (43).

#### Homology Modeling of hDAT

**Figure 1:**
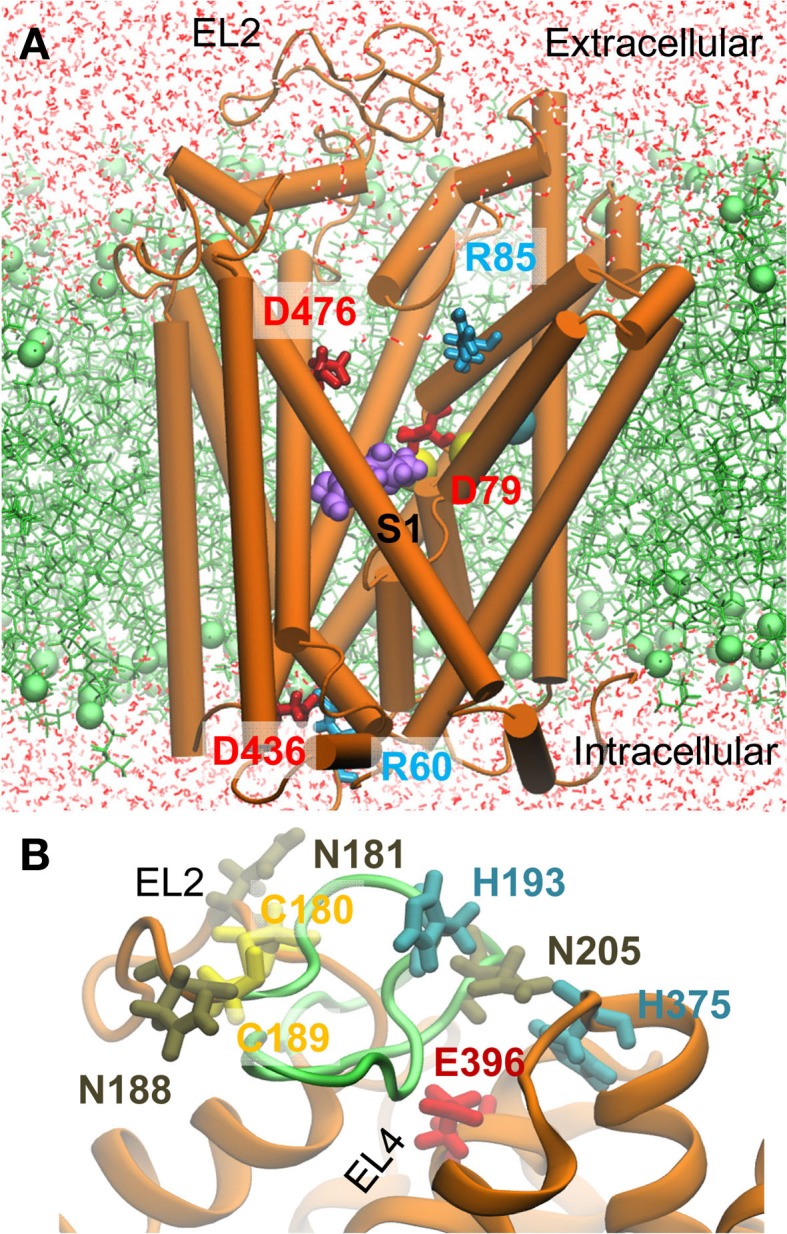
**Molecular dynamics (MD) setup for simulating the interaction of DAT with dopamine (DA), orphenadrine (ORPH), and amphetamine (AMPH)**. **(A)** A representative hDAT conformation observed in simulations. The hDAT OF*o* structure (orange) constructed using homology modeling based on dDAT [PDB: 4M48 (33)] is embedded into a POPC lipid bilayer (green). The substrate DA (purple) is initially placed in the most favorable binding site indicated by docking simulations. Water molecules are displayed in red lines. The cyan and yellow spheres represent the Cl^−^ and two Na^+^ ions resolved in the crystal structure. Two oppositely charged pairs of residues, R85-D476 (extracellular, EC) and R60-D436 (intracellular. IC), function as the putative EC and IC gates (counterparts of R30-D404 and R5-D369 in LeuT). **(B)** A closeup view of the modeled EL2 loop. The region (S190 to P212) whose homologous counterpart was unresolved in the crystal dDAT structure is shown in green. The labeled residues shown in stick representation were used as probes for model assessment (see the text).

Homology models for hDAT (Q58 to E598) in the OF*o* state were constructed using MODELLER (46) based on the dDAT crystal structure (33). The alignment of the two sequences was generated using Uniprot^2^. To model the human counterpart of the EL2 loop segment that was deleted in the crystal dDAT, we first considered two closely interacting cysteines (C148 and C157) at the end of the EL2 loop. The close positioning of these highly conserved cysteines in the resolved structure suggests that they formed a disulfide similar to that proposed for eukaryotic NSS family members (16, 47). Thus a disulfide bridge between their hDAT counterparts, C180 and C189, was adopted as a structural constraint in our homology modeling. One hundred homology models were constructed and that with the best (lowest MODELLER objective function) score was selected for further refinement and simulations (Figure 1). The quality of the modeled EL2 loop was assessed based on three criteria (16): (i) the three N-glycosylation sites N181, N188, and N205 were required to be exposed to the EC medium; (ii) C180 and C189 would form a disulfide bond; (iii) H193 and D206 (in EL2) would be in close proximity to H375 and E396 (in EL4) since a zinc ion is known to be coordinated by these four residues. The modeled EL2 loop adopted as the initial conformation satisfied all these three criteria (see Figure 1B). We note that during the course of simulations EL2 was highly flexible and disordered, and the putative Zn^2+^ binding site was transiently stabilized upon binding cations.

#### Docking Simulations

Docking simulations were performed with AutoDock (48) and Smina (49) using the energy-minimized hDAT OF*o* homology model as well as OF*o* conformers sampled during MD simulations. For each protein model/conformer, we performed our docking analysis under two conditions: in the presence of the sodium and chloride ions bound to hDAT and in their absence. Docking parameters for sodium and chloride were taken from the parameter library in AutoDock and fine-tuned to reproduce the binding pose of the antidepressant resolved in the crystal dDAT structure (33). The radius, vdW well depth, and effective charge were taken as 1.3 Å, 0.137 kcal/mol, and 1.0e, respectively, for Na^+^ ions; and 4.09 Å, 0.031 kcal/mol, and -1.0e, respectively, for Cl^-^ ion. For each system, 100 independent docking runs were performed using a Lamarckian genetic algorithm with default parameters (48), with the maximal number of energy evaluations set to 2.5 × 10^7^. The simulation box was divided into 112 × 112 × 126 grids with a spacing of 0.6 Å. The binding energy was estimated from the weighted average from multiple binding poses of the small molecule at a given site.

#### Parameterizations of Substrate/Drugs

Dopamine, AMPH, ORPH, or cocaine all carry +1 charge. Force field parameters for these small molecules were obtained from the CHARMM General Force Field (CGenFF version 0.9.7.1 beta) for drug-like molecules (50), using the web server ParamChem. The penalties associated with the use of the listed parameters for DA, AMPH, and ORPH were verified to be within acceptable limits (<10) such that no further refinement was required (51). In contrast, those predicted by ParamChem for cocaine had high penalties (>50). Instead, we used antechamber tool (52) implemented in AMBER (53) and MD simulations of cocaine binding were performed using AMBER (see Supplementary material).

#### MD Simulations

Unless otherwise stated explicitly, all MD simulations were performed using the NAMD2 software (54), adopting previous simulation protocol (25). MD simulation systems were set up using VMD (55). Two sodium ions, one chloride, and one cholesterol molecule resolved in the crystal structure (33) were included in the initial structure. The TM domain of hDAT OF*o* was inserted into the center of pre-equilibrated 1-palmitoyl-2-oleoylphosphatidyl choline (POPC) lipid bilayer, following previous approach (25). Fully equilibrated TIP3 waters were added to form a box of 104.6 × 104.6 × 150 Å^3^. Na^+^ and Cl^−^ ions corresponding to a 0.15 M solution were added to neutralize the system. The simulation box contained 1 hDAT, 196 POPC molecules, 88 Cl^−^ ions, 83 Na^+^ ions, 2 Zn^2+^, 4 cholesterol molecules, and about 29,100 water molecules, summing up to a total of over 140,000 atoms. One control system without drug/substrate and three additional systems in the presence of DA, AMPH, and ORPH were constructed. The ligand/drug were bound to the most favorable binding site identified by AutoDock, which is located in the vicinity of the primary substrate-binding site S1 (see Figure 1). To explore the existence of a possible secondary binding site S2 (56), an additional DA molecule was placed in the EC solution near the EC-facing vestibule of DAT.

CHARMM36 force field with CMAP corrections was used for hDAT, water, and lipid molecules (57–59). Prior to productive runs, each system was energy-minimized for 50,000 steps, followed by 0.5 ns constant volume and temperature (*T* = 310 K; NVT) simulations and a subsequent 4 ns Nosé–Hoover (60, 61) constant pressure and temperature (1 bar, 310 K; NPT) simulation, during which the protein was fixed and constraints on the POPC head groups were gradually released. Subsequently, the constraints on the protein backbone were reduced from 10 to 0 kcal/mol within 3 ns. Finally, the unconstrained protein was subjected to NPT simulations. For each of the four simulated systems, two independent MD runs of 100 ns were performed, designated as *apo-1* and *-2*, *DA-bound-1* and *-2*, *AMPH-bound-1* and *-2*, and *ORPH-bound*-*1* and -*2*.

#### Trajectory Analysis

VMD (55) was used to analyze the root mean square deviation (RMSD) of hDAT from its initial conformation, the root mean square fluctuations (RMSF) of the C^α^-atoms, the tilting angles of selected TM helices, the center-of-mass (CoM) distances between residue pairs of interest, and the formation/disruption of salt bridges. The orientational motions of TM1b (D79-N93) and TM6a (A308-L322) were evaluated based on the departure in the corresponding helical tilting angles from their original values. The stability of hDAT was assessed by evaluating the RMSD of the instantaneous conformers from the initial model, shown in Figure S1 in Supplementary Material. The RMSDs in eight different runs of 100 ns converged to an average value of 3.3 ± 0.6 Å. Departures from this average in some trajectories were mainly due to the high fluctuations of the EL2 loop. Figure S2 in Supplementary Material compares the RMSFs of C^α^-atoms in different forms (*apo*, and DA-, AMPH-, and ORPH-bound) of hDAT. RMSD calculations repeated by excluding the EL2 loop yielded an average RMSD of 2.0 ± 0.2 Å (Figure S1B in Supplementary Material).

### Experimental material and methods

#### Materials

HEK-293A cells (Invitrogen) were transfected with plasmid encoding DAT with an HA epitope inserted in the second EC loop (HA-DAT) (62) using Lipofectamine 2000 according to manufacturer’s protocol (Invitrogen). 3H-DA was from Perkin Elmer. ORPH and other chemicals were from Sigma.

#### DA Uptake Assays

Porcine aortic endothelial (PAE) cells with stable expression of hDAT were cultured as described previously (63). Cells were grown to confluence in 24-well dishes and treatments and assays were conducted in 37°C phosphate buffer saline (PBS) supplemented with 0.1 mM CaCl_2_, 1 mM MgCl_2_, and 10 mM glucose (PBS-CMG). Following 10 min uptake of ^3^H-DA, cells were washed twice with ice-cold PBS-CMG prior to lysis in 1% SDS and scintillation counting. For experiments (shown in Figure 8A), ORPH and ^3^H-DA were added simultaneously following 10 min pre-treatment with 10 μM cocaine as indicated. For kinetic assays (Figure 8B), ORPH was added 10 min prior to uptake.

#### Dopamine Transporter “HA Antibody Feeding” Endocytosis Assay

HEK cells with stable expression of HA-DAT were cultured on glass coverslips prior to HA antibody feeding assay (62). Cells were exposed to 1 μg/ml mouse anti-HA antibodies (HA11, Biolegend) in addition to treatment with vehicle (Veh), 100 μM AMPH, or 100 μM ORPH for 30 min prior to fixation in 4% paraformaldehyde. Cy3-conjugated donkey anti-mouse antibodies (2 μg/ml) were added for 1 h to label cell surface HA-DAT. Following 5 min permeabilization with 0.1% Triton-x-100, Cy5-conjugated donkey anti-mouse antibodies (1 μg/ml) were added to visualize endocytosed HA-DAT. Z-stacks of x–y confocal images were acquired using a spinning disc confocal imaging system based on a Zeiss Axio Observer Z1 inverted fluorescence microscope (with 63× Plan Apo PH NA 1.4) equipped with a computer-controlled spherical aberration correction unit, Yokogawa CSU-X1, Photometrics Evolve 16-bit EMCCD camera, and environmental chamber and piezo stage controller and lasers (405, 445, 488, 515, 515, 561, and 640 nm), all controlled by SLIDEBOOK5 software (Intelligent Imaging Innovations, Inc.). Image acquisition settings were identical in all experiments. For quantitation, 3D images of eight random fields (each image containing typically 10–15 cells) from each condition were acquired through 561 nm (Cy3) and 640 nm (Cy5) channels. Quantitation of the amount of Cy3 (surface HA-DAT) and Cy5 (surface plus internalized HA-DAT) fluorescence was performed using the statistics module of SLIDEBOOK6. The background-subtracted 3D images were segmented using a minimal intensity of Cy3 or Cy5 fluorescence as a low threshold to obtain segment Masks #1 and #2. Mask #1 was subtracted from Mask #2 to obtain Mask #3 corresponding to voxels containing only IC Cy5 fluorescence (internalized HA11 complexes with HA-DAT). The ratios of integrated intensities of Mask #3 to Mask #1 were calculated to determine the apparent extent of DAT internalization.

## Results

### PMF studies identified eight repurposable drugs for DAT, two of which are further supported by 3D similarity

**Figure 2:**
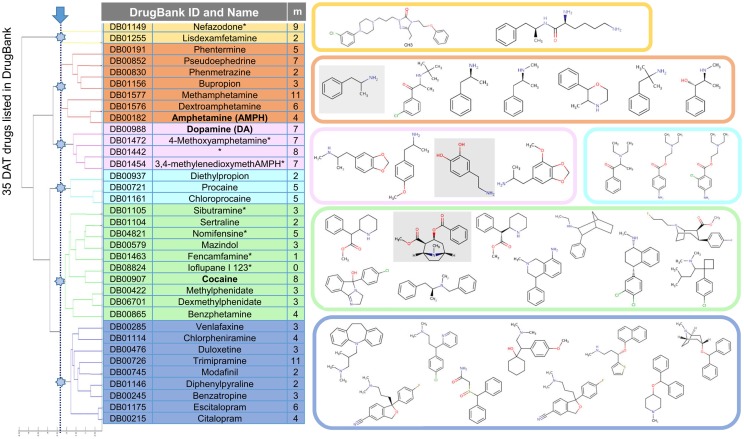
**Drugs/substrates listed in DrugBank v3.0 to interact with DAT, clustered in six groups based on their structural and chemical similarities**. Membership of each cluster is listed, along with the structures (color-coded). Three substrates/drugs on which we focus, dopamine, amphetamine (AMPH), and cocaine, are highlighted in gray boxes on the right. The table contains, in addition to the DrugBank identifiers (first column) and names (second column; with asterisks indicating illicit or withdrawn drugs that have been removed from further analysis), the number of targets (third column) listed (in DrugBank) for each drug.

We first examined the largest interconnected graph in DrugBank v3.0 (45) to generate clusters of drugs and focused on a cluster that is predominantly composed of drugs listed to interact with DAT. Figure 2 displays those drugs (35 of them), grouped into six clusters based on their 3D-structural and chemical characteristics [determined using OpenEye OMEGA (42) and Shape]. Of these, eight are illicit or withdrawn, which reduced the FDA-approved set to 27.

As a second step, we considered the bipartite graph composed of 1,413 FDA-approved drugs and 1050 protein targets contained in DrugBank to obtain 74 potentially repurposable drugs. These were inferred from the top 10 predictions in 10^5^ parallel PMF calculations (39, 41) repeated with different random/seed numbers. The resulting drugs are listed in Table S1 in Supplementary Material.

The combined set of 101 drugs was further subjected to agglomerative hierarchical clustering based on their latent (PMF-derived) vectors. We repeated our analysis with three different distance metrics, Euclidian, cosine, and city-block. In each case, among the resulting clusters, those enriched in known DAT drugs were selected (see Figure S3 in Supplementary Material) and further examined to sort those repurposable drugs commonly identified with different metrics. This led to eight hits: fluoxetine, levomilnacipran, milnacipran, desvenlafaxine, ORPH, ephedrine, atomoxetine, and protriptyline.

**Figure 3:**
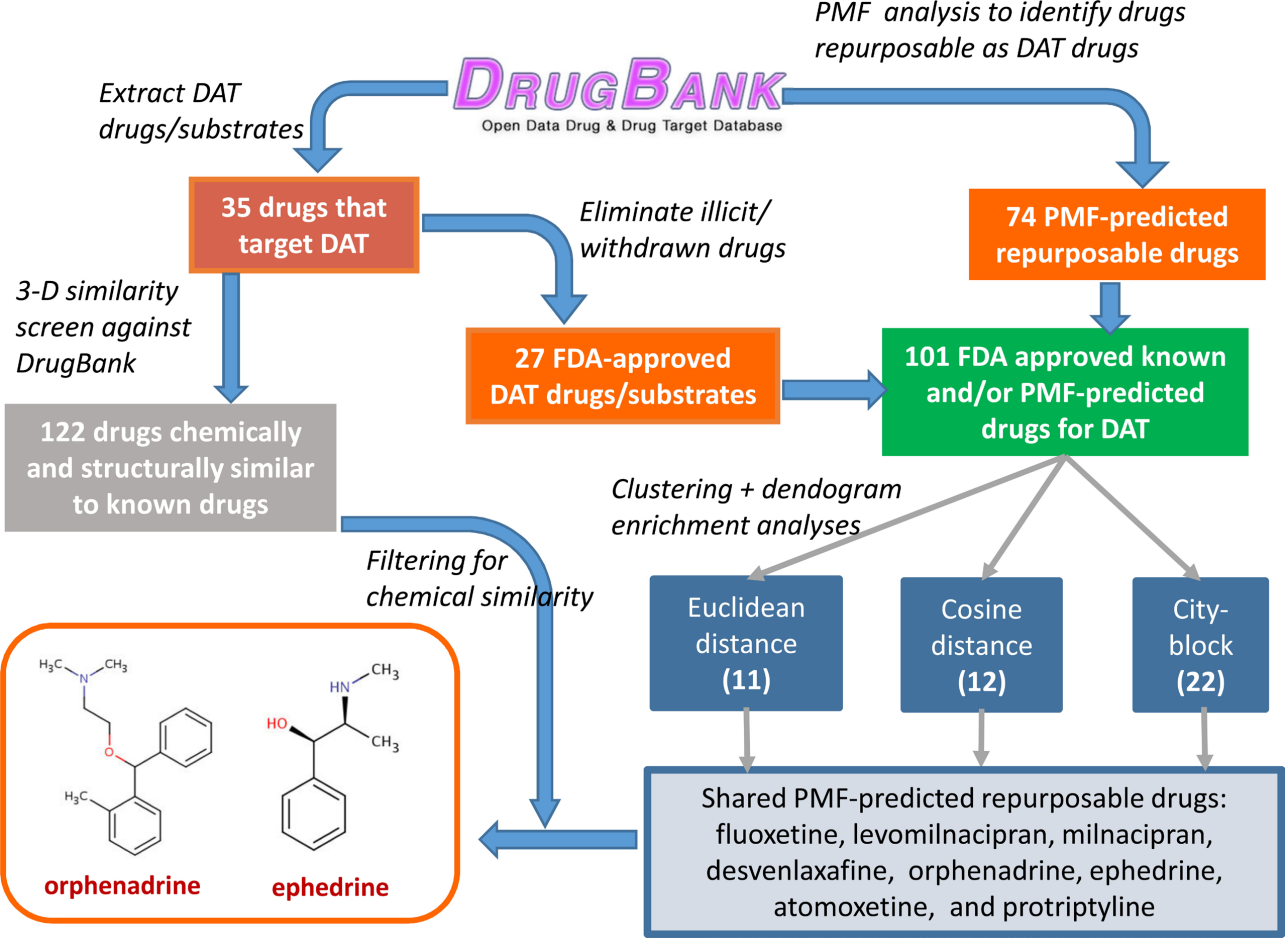
**Flow diagram for the consolidation of repurposable drugs for DAT**. A set of 101 drugs, composed of 27 FDA-approved drugs listed for DAT in DrugBank, and 74 repurposable drugs predicted by the PMF analysis (Table S1 in Supplementary Material), are grouped in clusters, based on their PMF latent vector similarities. Those clusters enriched in known drugs are examined to extract among repurposable drugs eight, consistently detected by different distance metrics (Euclidian, cosine, and city-block). Screening of these drugs against the subset of 122 drugs identified by OpenEye screening to be structurally and chemically similar to known drugs distinguishes two, ORPH and ephedrine (followed by desvenlafaxine, not shown). See the text for details. The highest scoring drug, ORPH, is selected for computational and experimental investigation.

Finally, these hits were further filtered based on their 3D fingerprint similarity to 122 drugs identified to be structurally and chemically similar to 35 original drugs, using a ComboScore threshold of 1.5 in OpenEye. This filtering procedure finally led to ephedrine and ORPH as two compounds that met both the PMF and 3D similarity criteria. ORPH, an anticholinergic agent and anti-PD drug (45), which yielded the highest score, has been selected for further tests/validation with the help of molecular computations and experimental assays. A schematic description of the overall procedure is provided in Figure 3.

### Docking analysis identifies a common high affinity binding pocket near the primary substrate-binding site S1

**Figure 4:**
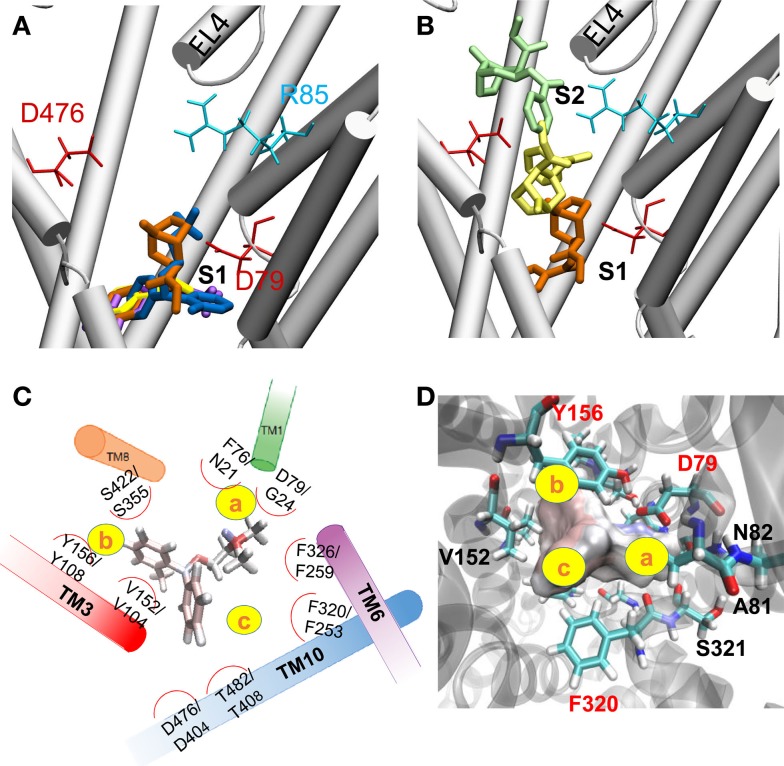
**Substrate/drug binding to hDAT outward-facing open (OF*o*) state**. **(A)** The most favorable binding site identified for DA (magenta stick), AMPH (yellow stick), ORPH (dark blue stick), and cocaine (orange stick). The site is broadly equivalent to the nortriptyline binding site resolved in dDAT, in the close vicinity of the substrate-binding site S1. **(B)** Alternative cocaine binding sites identified by AutoDock. Three poses representative of three clusters are shown, with docking energies varying from -6.6 (orange) to -5.5 (yellow), to -4.8 kcal/mol (green). These binding sites span the entire EC vestibule from the entrance (near S2) to the S1 site. **(C)** Comparison of the binding geometry of ORPH in hDAT, and that of Leu in LeuT. All residues that are within 3 Å distance from ORPH (based on atom-atom contacts) are listed along with their LeuT counterparts (written as hDAT/LeuT residues). **(D)** The residues coordinating the binding of ORPH at site S1 are distributed in three subsites, labeled a–c. More detailed view of the coordination geometry of ORPH near S1, displayed from two different perspectives. ORPH is shown in surface representation.

To identify the hDAT sites that potentially bind ligands or drugs, we performed docking simulations for DA, AMPH, ORPH, and cocaine. Computations performed in the presence and absence of Na^+^ and Cl^-^ ions near S1 led to comparable ligand binding clusters and affinities, indicating that the bound Na^+^ and Cl^-^ ions had a minor effect, if any, on ligand binding. This is presumably due to the fact that the ligand did not make direct contacts with these ions at the initial stage of binding. The most favorable binding site predicted by AutoDock was in all cases approximately halfway across the membrane in the EC vestibule (Figure 4A), equivalent to the nortriptyline binding site and close to the primary substrate binding site S1 (33). The binding poses of DA, AMPH, and cocaine were consistent with previous studies (13). Figures 4C,D show the coordination of ORPH, which displays similarities to that of Leu in LeuT (Figure 4C). For clarity, we divided the binding site into three subsites, a, b, and c (yellow circles). Subsite a is highly specific and presumably contributes to selective binding. In particular, F76 and D79 in hDAT (or their dDAT counterparts F43 and D46) coordinate the catecholamine DA binding, while N21 and G24 (in LeuT) contribute to the binding of amino acids, such as Leu or Ala. Sites b and c are amphipathic and composed of highly conserved residues.

All four of the investigated drugs/substrates, DA, AMPH, ORPH, and cocaine, as well as nortriptyline, are positively charged. Their amine groups were usually involved in attractive electrostatic interactions with D79. Our simulations clearly show the critical role of D79 in stabilizing the binding to site S1 (Figures S4C–F in Supplementary Material). Note that a switch in salt bridge involving R85, from R85-D79 to R85-D476, accompanies the translocation of DA from S2 to S1. Release of D79 upon D79-R85 dissociation opens the way to the association of D79 with DA.

### The S2 site at the upper EC vestibule may serve as a trap binding the ORPH or cocaine molecules that diffuse from the EC region

Drugs modulate the function of DAT in different ways [for a recent review see Ref. (2)]: some, such as cocaine, are not transportable; they inhibit substrate transport by stabilizing a certain conformation (e.g., OF state) and thus arresting the transport cycle; whereas others, such as AMPH, compete with DA; they may be transported as a substrate and stimulate the efflux of IC DA. Currently, there is no direct evidence in the literature whether ORPH binds DAT or not, or whether it is transportable or not. The above docking simulations show that it can bind and insert into the substrate-binding site S1 of hDAT, suggesting a competitive binding with other DAT substrates and drugs. The same simulations also showed, on the other hand, while the best docked models for DA and AMPH were exclusively confined to the close vicinity of site S1, those predicted for cocaine and ORPH varied over a broader range spanning an extended region between the EC vestibule and the site S1, including the vicinity of site S2. Figure 4B illustrates the alternative conformers predicted for cocaine.

As a further test, we explored the behavior of cocaine and ORPH molecules originally located in the EC region. MD simulations of binding from the EC region revealed the tendency of cocaine and ORPH binding to diffuse and settle near S2 [Figure S5 in Supplementary Material; secondary binding site proposed for Leu binding to LeuT (56)]. Therefore, while the S1 site of the original OF*o* structure is a high-affinity site, these relatively large ligands got trapped near S2. Further translocation to S1 was apparently hindered by tight interactions near S2. Simulations for DA, on the other hand, showed that DA proceeded to S1 after transiently binding S2 (Figure S4 in Supplementary Material). The inability of these two compounds to penetrate deeper into the binding pocket could affect, if not impair, the changes in the structure of the transporter that would otherwise be triggered upon ligand binding onto the primary site S1. This possibility is explored next by extensive MD simulations.

### DA and AMPH trigger the reconfiguration of hDAT upon binding, from the OF*o* to OFc* substate predisposed to substrate translocation; ORPH arrests it in the OFo* state

To further explore whether binding is succeeded by translocation, we constructed four MD simulation systems: one control without substrate/drug and three in the presence of DA, AMPH, or ORPH, docked to the S1 site of hDAT in the OF*o* conformation (Figure 1A). For each system, two independent 100 ns conventional MD runs were carried out to verify the reproducibility of the results.

**Figure 5:**
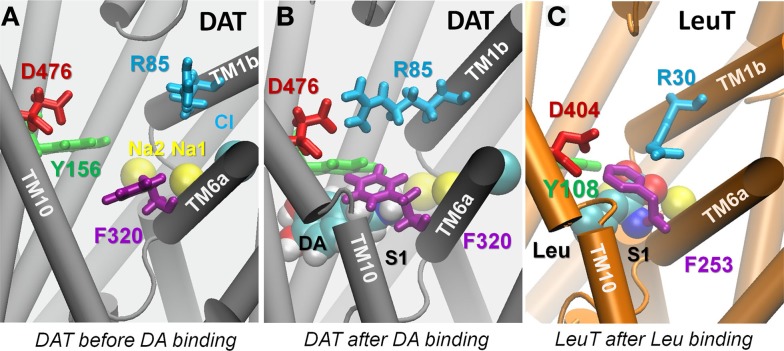
**Dopamine binding to site S1 prompts the closure of EC gate and dopamine-bound hDAT resembles Leu-bound LeuT**. **(A)** Substrate binding pocket before dopamine binding, typical of the outward-facing open conformation; **(B)** same pocket after dopamine (vDW format) binding. Isomerization of F320 (purple) brings its aromatic side chain on top of dopamine. Close association of Y156-F320 and salt bridging of R85-D476 designate the closure of the EC gates, indicating hDAT in its outward-facing closed state; and **(C)** crystal structure of LeuT with Leu bound to the S1 site (PDB: 2A65), in which the homologous EC gate residues orient in a similar pose to that shown in **(B)**. Snapshot in **(B)** is taken from 100 ns in *runDA-bound-1*.

**Figure 6:**
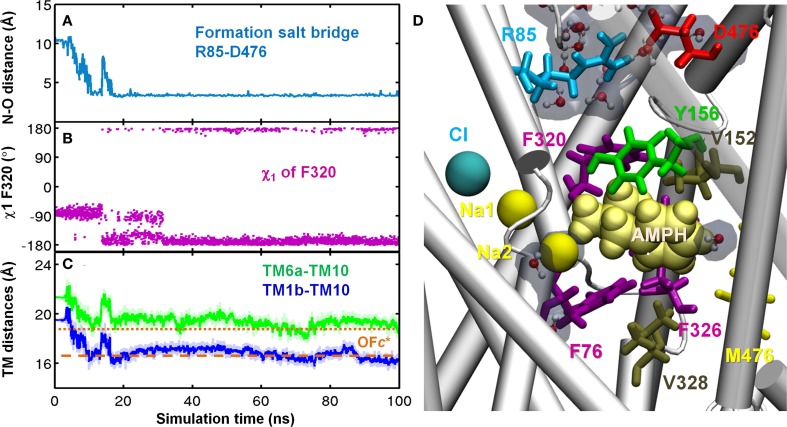
AMPH binding to site S1 facilitates hDAT structural transition from outward-facing open (OF*o*) to outward-facing closed (OFc*) state. Time evolutions of **(A)** N–O distance of R85-D476. Salt-bridge was formed around 20 ns; **(B)** χ_1_ of F320. F320 flipped on top of dopamine as χ_1_ stably changed from -80 ± 15° to 170 ± 15° around 35 ns; **(C)** CoM distances of EC-exposed TM segments TM6a–TM10 (green) and TM1b–TM10 (blue). **(D)** In the OFc* state, side view of the binding site for AMPH (yellow vDW) was dehydrated and occluded to both EC and IC region. Two hydrophobic layers F320-V152 (upper) and F76-F326-V328 (lower) prevent water penetration from both sides. Water molecules in the EC and IC vestibules are shown in CPK format with semi-transparent cyan. Results were taken from *run* *AMPH-bound-1*. Transition from OF*o* to OFc* was also observed in *AMPH-bound-2*.

Binding of either DA or AMPH to the S1 site was consistently observed to trigger significant conformational changes in the EC vestibule, which led to the OFc* state. The OF*o* and OFc* states were distinguished by three major criteria (24): opening/closure of the outer and inner EC gates, inter-helix packing, and hydration pattern of both the EC and IC vestibules. First, the EC gates were observed to close within tens of nanoseconds (Figures 5 and 6; Figures S6 and S7 in Supplementary Material). In particular, the salt-bridge of R85-D476 that serves as the outer EC gate spontaneously formed after DA- (Figure 5) or AMPH-binding (Figure 6); and the F320 side chain underwent a rotational isomerization, from χ_1_ = -80 ± 15° to -170 ± 15° (Figure 6B; Figure S7B in Supplementary Material), which enabled its association with Y156, forming the inner EC gate that further sealed the substrate from the EC environment. The closure of these two EC-gates R85-D476 (R30-D404 in LeuT) and Y156-F320 (Y108-F253 in LeuT) upon the binding of DA or AMPH (Figure S6 in Supplementary Material) to the S1 site is in striking similarity to the sequence of events observed in our previous investigation of Ala binding to LeuT (25).

**Figure 7:**
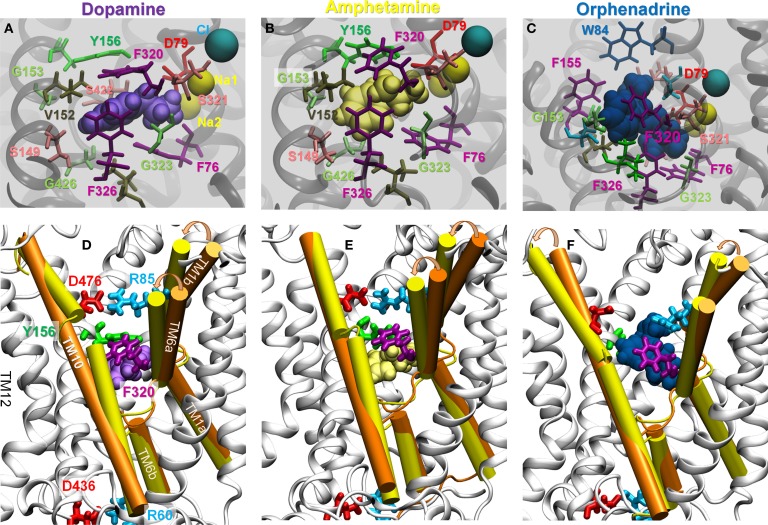
**Specific interactions of hDAT with DA (left), AMPH (middle), and ORPH (right) and accompanying structural changes in hDAT**. Binding of either DA or AMPH leads to hDAT reconfiguration from the OF*o* to OFc* state; whereas binding of ORPH has no such effect, and the OF*o* conformation is further stabilized. **(A–C)** Illustrate the coordination geometry of **(A)** DA, **(B)** AMPH, and **(C)** ORPH. Alignment of the initial OF*o* hDAT (orange) with the MD-relaxed hDAT (yellow) in the presence of **(D)** DA; **(E)** AMPH; and **(F)** ORPH. For clarity, only TM1, TM6, and TM10 helices are highlighted in cartoon and MD-relaxed structures are shown in white ribbon. Results are taken from the last 100 ns MD simulations in each case.

Second, the binding of DA or AMPH prompted an inward tilting of TM1b (10–15°) and TM6a (2–10°) segments toward the center of the EC vestibule (Figures 6C and 7; and Figure S7C in Supplementary Material). The inter-helical packing geometry of the EC vestibule reached values typical of OFc* state within 100 ns MD simulations (Figures 6C; Figure S7C in Supplementary Material). Notably, the DA- or AMPH-bound hDAT resembles significantly the Leu-bound LeuT in the OFc* state (Figures 5 and 7), which is predisposed to substrate translocation and release as confirmed in earlier work (24, 25).

Third, in contrast to the OF*o* state where there is a continuous water occupancy in the EC vestibule, in the OFc* state, the site S1 is minimally hydrated: it contains only 3–5 water molecules that assist in coordinating one of the two co-transported sodium ions (designated as Na2) and the substrate (Figure 6D; Figure S7D in Supplementary Material). Two layers of hydrophobic interactions, one on each side of the binding site, seclude the binding site from water penetration from either the EC and IC region: F320-V152 (upper) and F76-F326-V328 (lower). Overall, similar residues coordinate the binding of DA or AMPH in the OFc* state. These include F76 and D79 on TM1; S149, V152, G153, and Y156 on TM3; F320, S321, F326, and V328 on TM6; and S422 and G426 on TM10 (Figures 7A,B).

While DA- and AMPH-bound hDAT spontaneously proceeded from OF*o* to OFc*, evidenced by these three distinctive properties, a completely different behavior was exhibited by ORPH-bound hDAT. ORPH was coordinated by W84 (TM1b) and F155 (TM3) in addition to other residues that also coordinate DA and AMPH (Figure 7). A major difference was that in contrast to DA and AMPH, no significant closure of the EC vestibule was observed in two independent simulations of ORPH-bound hDAT (Figures S6 and S7 in Supplementary Material); essentially hDAT maintained its OF*o* conformation, with continuous water occupancy at the EC vestibule. The EC-exposed TM6a displayed negligible change in its orientation and the TM10 segment underwent a ~5° outward tilting to even further enlarge the EC vestibule rather than contracting it, presumably to accommodate the larger size ORPH. Notably, TM1b (exposed to the EC region) underwent a ~5° inward tilting, although this reorientation remained ~5° smaller compared to that observed in DA-bound or AMPH-bound hDAT. ORPH wedged between F320 (TM6a) and Y156 (TM3). Due to its bulky size, F320 was not able to flip over the top of ORPH to seclude it from the EC region, as observed in AMPH-bound and DA-bound hDAT (Figures 7A–C), but remained on the side; the F320-Y156 distance maintained its typical value (12 ± 1.0 Å) in the OF*o* conformation, which is ~4 Å larger than that observed in the OFc* state (Figure S6 in Supplementary Material).

Given that the DAT conformational changes conducive to the translocation of substrate could not occur in the ORPH-bound transporter, we propose that like cocaine, ORPH is not likely to be transported. This is due to both its bulky size which hinders the closure of the EC gating pairs D476-R85 and F320-Y156 (compare in Figures 7D–F) and impedes the concerted inward tilting of the EC-exposed TM helices. Our conjecture is supported by docking simulations of ORPH, cocaine, DA, and AMPH conducted with the hDAT in the OFc* conformation, obtained by the present MD simulations. No ORPH or cocaine molecule was found to bind the EC vestibule in the OFc* state, while DA and AMPH exhibited significant increase of binding affinity to the S1 site (Table S2 in Supplementary Material). Furthermore, neither DA nor AMPH were able to bind the S2 site in the hDAT OFc* conformation, suggesting that the site S2 (56) is a transient substrate-binding site accessible when hDAT is in the OF*o* state only (see Figure S4 in Supplementary Material).

Simulations initiated by placing the ORPH and cocaine more than 20 Å away from the site S1 (Figure S5 in Supplementary Material) also showed that the pair F320-Y156 maintained its typical distance of 13.5 ± 0.5 Å of the OF*o* conformation over the entire simulations. The EC salt-bridge of R85-D476 intermittently formed in ORPH-bound OF*o*, preventing the ORPH from penetrating further toward S1. The formation of this salt bridge was not sufficient, however, for the closure of the EC vestibule. Therefore, the transporter remained arrested in OFo* state.

### Substrate transport experiments confirm ORPH as competitive inhibitor

**Figure 8:**
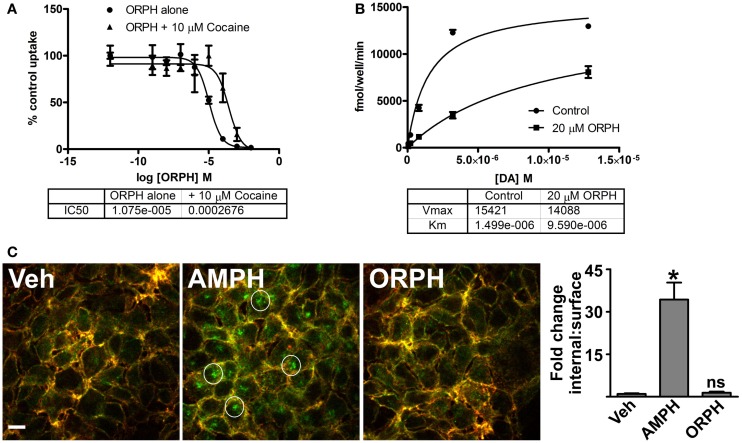
**(A)** Competitive inhibitory properties of ORPH confirmed by experiments. Uptake of ^3^H-DA by PAE cells expressing human DAT was measured in the presence of the indicated concentration of orphenadrine (ORPH) alone or following 10 min pre-treatment with 10 μM cocaine. Means of three replicates ± SEM were plotted as a percentage of the uptake remaining from lowest ORPH concentration in respective group (either alone or with cocaine). ORPH concentration for 50% inhibition of dopamine uptake (IC50) was calculated. **(B)** Uptake of the indicated concentrations of ^3^H-DA by PAE cells expressing hDAT was measured in the presence of no drug (control) or following 10 min pre-treatment with 20 μM ORPH. Mean ± SEM of three replicates were plotted and Michaelis–Menten non-linear regression analysis was performed, providing transporter dopamine affinity (*K*_m_) and capacity (*V*_max_) for each condition. **(C)** HEK cells expressing HA-DAT were treated for 30 min with water vehicle (Veh), 100 μM amphetamine (AMPH), or 100 μM orphenadrine (ORPH) and were subjected to the “HA antibody feeding” assay as described in section “Materials and Methods” prior to imaging by confocal fluorescence microscopy. Surface transporters appear red and green (yellow), while intracellular transporters appear in green only. Examples of endocytosed HA-DAT (green) are circled. Representative images from three replicates are shown. The fold change in the ratio of IC:surface transporters was determined by quantitative analysis of surface and IC signals as described in section “Materials and Methods.” Values are the means of eight determinations and error bars are standard deviations. Significant difference from vehicle (*p < 0.05) was determined by one-way ANOVA followed by the Tukey post-test for multiple comparisons.

To determine whether ORPH affects DAT function, we measured uptake of ^3^H-DA by PAE cells that express the human DAT. ORPH caused a dose dependent decrease in DA uptake with an IC50 of ~10 μM (Figure 8A). Cocaine, a competitive inhibitor of DA transport, reduces inhibition by other competitive inhibitors but not non-competitive (allosteric) inhibitors. In the presence of 10 μM cocaine, the IC50 of ORPH increased more than 20-fold, suggesting that ORPH, like cocaine, is a competitive DAT inhibitor (Figure 8A). While DAT inhibitors like cocaine inhibit DAT function by blocking substrate binding, the DAT substrate AMPH can also modulate both the affinity and the capacity for DA transport by engaging IC signaling pathways that alter the expression of the transporter at the cell surface (10, 64). To examine the effect of ORPH on DAT substrate affinity and capacity, we performed ^3^H-DA uptake experiments in the presence of 20 μM ORPH. ORPH decreased the affinity of DA for the transporter by nearly 10-fold, with little change in transport capacity (Figure 8B). These results suggest that ORPH inhibits DA transport by interfering with DA binding as opposed to downregulating the plasma membrane transporter.

To assess more directly whether ORPH affects the plasma membrane expression of the DAT, we assessed transporter internalization using confocal fluorescence microscopy. DAT containing an HA epitope inserted in the second EC loop (HA-DAT) will carry bound HA antibodies from the plasma membrane to endosomes, allowing efficient visualization and quantitation of endocytosis (62). HEK cells expressing HA-DAT were incubated for 30 min in the presence of anti-HA antibodies along with vehicle, AMPH, or ORPH treatment (Figure 8C). Following fixation, cell surface HA-DAT was detected by incubation with Cy3-conjugated secondary antibodies (red) prior to permeabilization, and endocytosed transporter was detected by incubation with Cy5-conjugated secondary antibodies (green) after permeabilization. HA-DAT accumulated in IC compartments following treatment with AMPH, while cells treated with ORPH resembled the control condition with predominantly plasma membrane transporters. All together, these results indicate that the actions of ORPH are similar to that of the DAT competitive inhibitor cocaine, as opposed to the DAT substrate AMPH.

## Discussion

The major findings in this study may be summarized as follows: (i) the identification and validation of ORPH as a repurposable drug for DAT, supported by both computations and experiments, (ii) the elucidation of the mechanisms of interaction of hDAT with substrates (DA and AMPH) and blockers (ORPH and cocaine), pointing to the critical role of selected residues such as D79, the salt-bridge R85-D476, and aromatic pairs F320-Y156 serving as outer and inner EC gates, respectively, (iii) the demonstration of the contrast between the conformational changes of hDAT triggered by these two groups of compounds, consistent with their distinctive modulation on DAT function: the former two spontaneously prompt structural transitions toward facilitating DA/AMPH translocation, the latter two arrest hDAT in the outward-facing open (OFo*) state, and (iv) the primary binding site S1 is a high affinity site for all four examined compounds. However, access to this site from the EC region varies. While the DA and AMPH unambiguously locate this site, cocaine/ORPH may bind multiple sites in the EC vestibule. In particular, the site S2 emerges as a first stop for binding EC substrates and ORPH and cocaine exhibit a tendency to get trapped therein due to their larger sizes and tighter interactions.

Previous structure-based studies of DAT-substrate/drug interactions used LeuT, which shares 22% sequence identity with hDAT, as template. Despite the low sequence similarity, the conservation of the fold and local packing geometry near the substrate-binding site permitted to gain insights into DAT conformational changes involved in cocaine binding and DA translocation (13, 18–20, 65–69). Here, for the first time, we used as template, the recently resolved dDAT structure (33), which shares >50% sequence identity with hDAT, and we modeled the EL2 loop unresolved in the fruit fly orthologs using as constraint data from previous cross-linking studies (16, 47) (Figure 1). The new model yielded many results corroborating previous findings, highlighting the structural and functional features conserved among LeuT fold family members in support of the use of LeuT as a template in earlier studies. For example, the R30- D404 gate of LeuT is replaced by R85-D476, or Y108-F253 of LeuT is replaced by Y156-F320 in hDAT. Recent work showed that DAT R85D mutant had a complete loss of function, which was restored by the compensating mutation R85D/D476R (68). In addition to such generic features, this study provides time-resolved full-atomic data and functional information specific to hDAT in the presence of DA, AMPH, cocaine, and ORPH. Yet, we note that due to the flexibility of the modeled EL2 loop, the putative Zn^2+^ binding pocket may be destabilized in the simulations. Further refinement of the EL2 loop using more structure constraints gleaned from experimental studies (16) may help.

Our docking simulations suggest that the most favorable binding site for cocaine and ORPH resides in the close proximity of the primary binding site S1, where it may compete with substrate (Figure 4A). These results are consistent with previous studies (13, 70) as well as the binding of antidepressant nortriptyline onto dDAT (33). In contrast, tricyclic antidepressants desipramine and clomipramine were crystallographically found to bind LeuT non-competitively at a site equivalent to site S2 (22, 71). A previous computational study of hDAT (20), modeled based on LeuT, proposed an allosteric triggering of DA release from S1 through the binding of another DA to S2. While the existence of S2 [as either a high affinity (72) or low-affinity site (23) for substrate binding] is well-established in LeuT and other NSS family members, such as DAT, SERT, and NET (71, 73, 74), crystal structural and functional analyses cast doubts on its relevance to the allosteric regulation of substrate release (23). The present MD simulations suggest that site S2 may be a first stop for the recognition and transient binding of DA from the EC medium (Figure S4 in Supplementary Material), and it may act as a trap precluding the effective penetration of the blockers, ORPH and cocaine, into the inner EC vestibule (Figure S5 in Supplementary Material). ORPH, like cocaine, may be involved in multiple binding pauses/interactions in the EC vestibule, supporting the view that inhibition can occur through multiple binding mechanisms (73). The present docking studies as well as MD simulations clearly demonstrate that the mechanism of inhibition by ORPH is the stabilization of the OFo* conformation (Figure 7). Furthermore, this study suggests that the ligand binding affinity is state-dependent (see Table S2 in Supplementary Material).

In our simulations, D79 was noted to play an important role in coordinating the ligand binding to site S1 (Figures 4 and 7). This aspartate is conserved among neurotransmitter transporters, such as DAT, SERT, and human desipramine-sensitive NET that share the LeuT fold; it facilitates the recognition of catecholamines (compounds such as DA, serotonin, epinephrine, and norepinephrine, which contain a benzene group with two attached hydroxyls). Substitution of the counterpart D75 in hNET by alanine led to an almost complete loss of NE uptake (75). Likewise, mutations of D79 to alanine, glycine, or glutamate significantly reduced DA uptake and the affinity for tritium-labeled cocaine analog (75). The fact that D79E also reduces DA uptake suggests that not only the electrostatic potential but also the size of amino acid at position 79 is important in mediating DA translocation. The reduced DA uptake in these mutants was attributed to the reduced ability to recognize DA or to the inability of the transporter to efficiently transport it after recognition (75).

Comparison of the effect of ORPH on hDAT structural dynamics to that of the substrates DA and AMPH reveals striking differences. DA and AMPH practically exhibit the same effect (compare Figure 6 for AMPH and Figure S7 in Supplementary Material for DA; or the middle two panels in Figure S6 in Supplementary Material; or the panels for DA and AMPH in Figure 7). Like DA, AMPH can translocate from the EC to the IC region mediated by DAT (76). In addition, AMPH may induce non-vesicular release of DA and trigger the subsequent efflux of DA back to EC region (76), thus increasing the EC DA levels in motivational and reward areas of the brain. Currently, it remains elusive how AMPH triggers non-vesicular release of DA and then the efflux of DA. It has been proposed that AMPH causes DAT-mediated DA efflux via two independent mechanisms: (76) one slow process consistent with transporter mechanism and the other rapid process through channel-like mode of DAT. In this study, we observed that binding the EC AMPH led to hDAT structural transition from OF*o* to OFc* state, which broadly resembles that induced by the binding of EC DA. We suggest that binding of the EC AMPH may not directly trigger the DA efflux. Further studies investigating the effect of AMPH binding to the IC-exposed domains of hDAT, including in particular the N-terminal segment, are needed to elucidate the molecular basis of DAT efflux elicited by AMPH.

## Conclusion

In this study, we utilized QSP and computational biology methods and molecular biology experiments to study the structure- and time-dependent mechanisms of interactions of known (cocaine, DA, and AMPH) and predicted (ORPH) drugs/substrates with human DAT (hDAT). First, by taking into consideration both structural similarities (compared with known agonists/antagonists) and functional patterns (from drug–protein interacting data, analyzed by machine learning method), we identified ORPH as a repurposable drug that might bind DAT and alter/inhibit its activity. Examination of the binding properties of ORPH, AMPH, DA, and cocaine, revealed two distinct modulation effects. While ORPH like cocaine inhibits hDAT function by stabilizing the OFo* state (or preventing the closure of the EC vestibule) and thereby arresting the transport cycle, binding of AMPH or DA triggered cooperative conformational changes, including TM helices rearrangements, conducive to substrate transport.

Computational prediction of repurposable drugs became an important research goal in recent years, after the pioneering work of the Schoichet laboratory (35). The method we introduced to this aim is based on the target similarities between drugs (40, 41). While such machine-learning algorithms provide new hypotheses, it is essential to thoroughly examine them by additional molecular simulations and by experiments/assays to test and establish the effects of the repurposable drugs. Here, statistical analyses and chemical similarity-based screens for selecting from hits (Figures 2 and 3), docking computations (Figure 4), MD simulations (Figures 5–7), DA-uptake, and DAT internalization assays (Figure 8) showed the ability of ORPH to competitively bind DAT, prevent closure of EC gates, and cause a reduction in DA uptake and in DAT endocytosis.

Design of transporter blockers is the most commonly applied strategy for antidepressant therapy (38), and notable advances are currently made in search for novel ligands that allosterically modulate DAT function (77). In principle, from pharmacological point of view, it is desirable to have a compound that prevents the binding of cocaine, while retaining the DA-transport function of DAT. Our experiments showed a decrease in DA reuptake in the presence of ORPH, without significant downregulation in the transport capacity of the transporter. Further studies with ORPH and other repurposable drugs (e.g., ephedrine, desvenlafaxine) could be worth pursuing to identify compounds that optimally interfere with cocaine binding while leaving DA binding and transport properties unaffected. Furthermore, design of drugs that can potentially perturb hDAT interactions with regulatory proteins (78) or altering hDAT trafficking (79) might be useful in developing new approaches for regulating hDAT function and DA neurotransmission *in vivo*.

## Author Note

After acceptance of our manuscript, the crystal structures of dDAT resolved in the presence of cocaine, dopamine, and amphetamine has been published (80). Notably, the binding poses of these drugs as well as their binding sites on dDAT show close similarities to those predicted by our simulations for substrate/drug bound hDAT in the OFo state (Figure 4A). Our MD simulations further revealed that binding of AMPH or dopamine to the site S1 promotes the closure of the outward-facing hDAT and stabilizes the OFc* state (Figures 5 and 6).

## Conflict of Interest Statement

The authors declare that the research was conducted in the absence of any commercial or financial relationships that could be construed as a potential conflict of interest.

## Supplementary Material

The Supplementary Material for this article can be found online at http://journal.frontiersin.org/article/10.3389/fneur.2015.00134/abstract

## Footnotes

^1^http://www.eyesopen.com

^2^http://www.uniprot.org
