# Supplementary material for: Insights into the Modulation of Dopamine Transporter Function by Amphetamine, Orphenadrine, and Cocaine Binding
Source: Front Neurol. 2015 Jun 9;6:134. doi: 10.3389/fneur.2015.00134 (PMC4460958; doi:10.3389/fneur.2015.00134)
Supplement: Supplementary file 1 [file Presentation_1.PDF]

Supplementary Material

Insights into the modulation of dopamine transporter function by amphetamine, orphenadrine and cocaine binding

Mary Hongying Cheng [a], Ethan Block[b], Feizhuo Hu[a,c], Murat Can Cobanoglu [a], Alexander Sorkin[b], Ivet Bahar[a,*]

Departments of Computational & Systems Biology [a] and Cell Biology[b], School of Medicine, University of Pittsburgh, Pittsburgh, PA 15213, USA; [c]Department of Pharmacology and Pharmaceutical Sciences, School of Medicine, Tsinghua University, Beijing, 100084, China.

*To whom correspondence should be addressed:

Dr. Ivet Bahar
Distinguished Professor and John K. Vries Chair
Computational & Systems Biology
School of Medicine, University of Pittsburgh
3064 Biomedical Science Tower 3
3501 Fifth Avenue, Pittsburgh, PA 15213

Voice: 4126483332 - Fax: 4126483163

bahar@pitt.edu; http://www.ccbb.pitt.edu/Faculty/bahar/

Supplementary Text

MD simulations of cocaine- and ORPH-binding to hDAT OF*o* using AMBER

Parameters for cocaine and ORPH were generated using Antechamber Tool (1) of AMBER for small chemicals. Two simulation systems (cocaine_OF*o* and ORPH_OF*o*) were constructed, in which the respective small molecules were placed near the transporter extracellular entrance, 25 Å away from the substrate binding site S1. MD simulations were performed using AMBER (2) with AMBER ff12SB force field for the protein and lipid11 force field for the lipids. Simulations were performed following standard procedures, including 1,000 steps of energy minimization followed by Nosé-Hoover (3, 4) NPT simulations. Restraints on the protein backbone were gradually reduced and finally unconstrained MD simulations were performed for a total of 100 ns.

Supplementary Tables

Table S1. DrugBank Compounds that may Potentially Target DAT

#	DBID	FullName	#	DBID	FullName
1	DB01173	Orphenadrine	38	DB00934	Maprotiline
2	DB00543	Amoxapine	39	DB01159	Halothane
3	DB00321	Amitriptyline	40	DB01110	Miconazole
4	DB01364	Ephedrine	41	DB00898	Ethanol
5	DB00193	Tramadol	42	DB00289	Atomoxetine
6	DB00805	Minaprine	43	DB06700	Desvenlafaxine
7	DB06204	Tapentadol	44	DB00449	Dipivefrin
8	DB00514	Dextromethorphan	45	DB00292	Etomidate
9	DB01151	Desipramine	46	DB00344	Protriptyline
10	DB01242	Clomipramine	47	DB00811	Ribavirin
11	DB04855	Dronedarone	48	DB01233	Metoclopramide
12	DB00622	Nicardipine	49	DB00904	Ondansetron
13	DB00215	Citalopram	50	DB00202	Succinylcholine
14	DB00661	Verapamil	51	DB00674	Galantamine
15	DB00568	Cinnarizine	52	DB00570	Vinblastine
16	DB01142	Doxepin	53	DB00393	Nimodipine
17	DB01136	Carvedilol	54	DB08918	Levomilnacipran
18	DB04896	Milnacipran	55	DB00780	Phenelzine
19	DB00458	Imipramine	56	DB00747	Scopolamine
20	DB06148	Mianserin	57	DB00659	Acamprosate
21	DB01579	Phendimetrazine	58	DB08896	Regorafenib
22	DB00370	Mirtazapine	59	DB00679	Thioridazine
23	DB00768	Olopatadine	60	DB00280	Disopyramide
24	DB01287	Dihydroergotoxine	61	DB00619	Imatinib
25	DB01267	Paliperidone	62	DB01064	Isoproterenol
26	DB00831	Trifluoperazine	63	DB00583	L-Carnitine
27	DB00786	Marimastat	64	DB00308	Ibutilide
28	DB00696	Ergotamine	65	DB00536	Guanidine
29	DB00920	Ketotifen	66	DB01239	Chlorprothixene
30	DB00909	Zonisamide	67	DB01618	Molindone
31	DB06637	Dalfampridine	68	DB00697	Tizanidine
32	DB00915	Amantadine	69	DB00472	Fluoxetine
33	DB00656	Trazodone	70	DB04844	Tetrabenazine
34	DB00540	Nortriptyline	71	DB08901	Ponatinib
35	DB00715	Paroxetine	72	DB00363	Clozapine
36	DB06707	Levonordefrin	73	DB01407	Clenbuterol
37	DB01049	Ergoloid mesylate	74	DB01221	Ketamine

(*) obtained by PMF of DrugBank v3.0 data. See Materials & Methods, and references 40 and 41 for more details.

Table S2. DAT State-dependent binding affinity to S1 site[(a)]

Substrate/drug	OF*o* (kcal/mol)	OF*c* (kcal/mol)
**DA**	-5.8±0.5	-7.0±0.5
**AMPH**	-5.0±0.5	-6.1±0.5
**ORPH**	-6.5±0.5	N/A
**Cocaine**	-6.2±0.6	N/A

[(a)] *using AutoDock. Mean values and standard deviations based on multiple runs (see Methods) are reported.*

Supplementary Figures

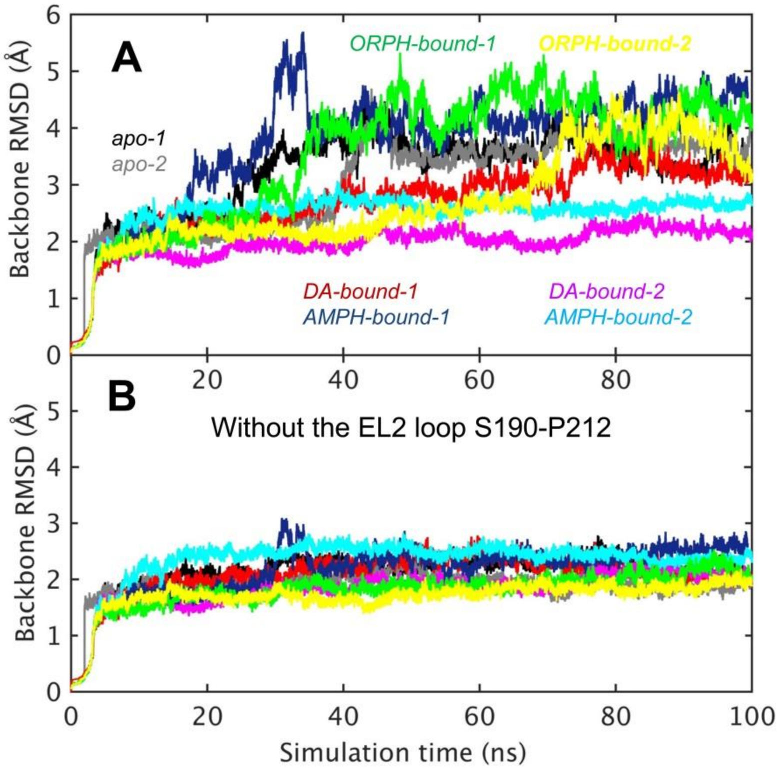

Figure S1. Time evolution of hDAT backbone RMSD with respect to the initial OF*o* conformation. The RMSDs are shown for (**A**) the overall hDAT structure (Q58 to E598); and (**B**) the hDAT structure excluding the EL2 loop residues S190-P212. In two runs (*DA-bound-2* and *AMPH-bound-2*), one sodium ion migrated from the EC region into the pocket between EL2 and EL4 segments, similar region to the proposed Zn^{2+} binding site (5). The binding of sodium ion resulted in a plateau at 2.2–2.4 Å (*pink* and *cyan* lines in **panel A**), which is comparable to the RMSD observed in a recent study of Zn^{2+} binding to hDAT (5).

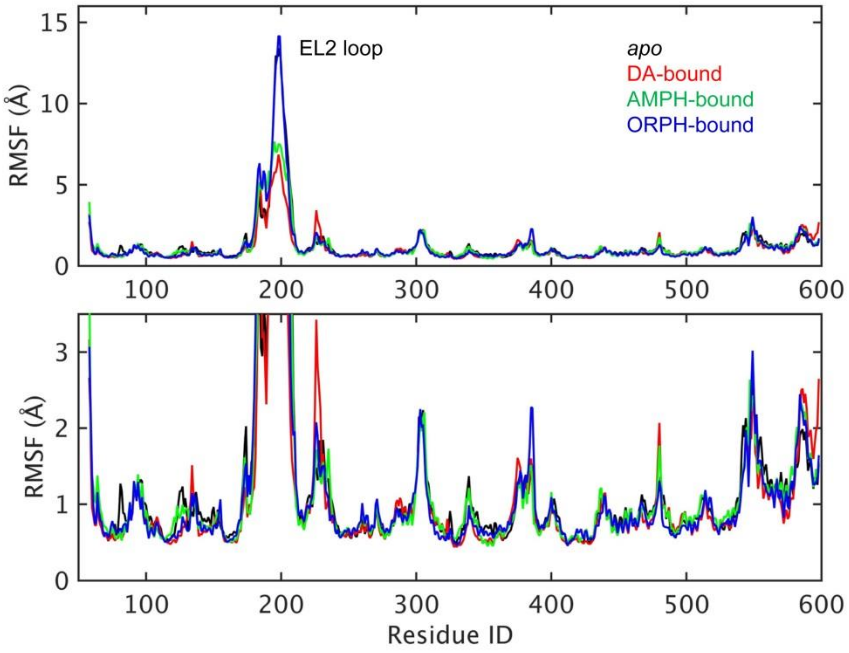

Figure S2. Root-mean square fluctuations (RMSFs) of hDAT C^{α} atoms in the simulations. Results are plotted and averaged from the two 100 ns simulations of *apo* (*black*; *apo-1* and *-2*), DA-bound (*red; DAT-bound-1* and *-2*), AMPH-bound (*green; AMPH-bound-1 and AMPH-bound-2)*, and ORPH-bound (*blue*; *ORPH-bound-1 and ORPH-bound-2*) hDAT. The *top* and *bottom* panels show the same results, with different scales to allow for clearer visualization. Note that the EL2 loop (S190 to P212) (**Figure 1B**) was very flexible.

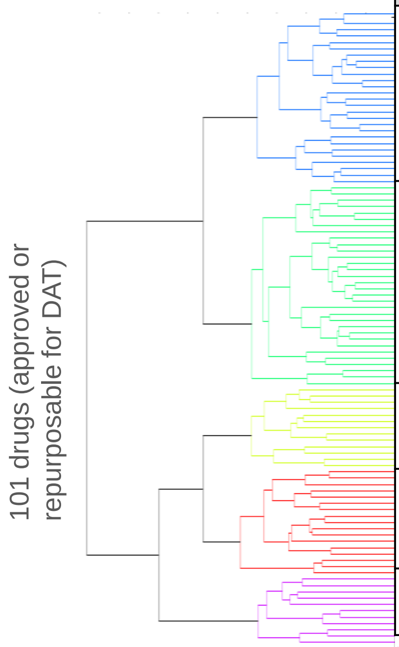

	N_K = # of known drugs	N_P = # of Predicted drugs	Enrichment $(N_K/N_P)_{observed}$ $/(N_K/N_P)_{expected}$
	6	22	0.273/0.365 = 0.748
	20	12	1.667/0.365 = **4.567**
	1	12	0.083/0.365 = 0.227
	0	17	0
	0	11	0
**Total**	**27**	**74**	**0.365** *(expected)*

ID	Repurposable drug name
DB00193	Tramadol
DB00289	**Atomoxetine**
DB00344	**Protriptyline**
DB00472	**Fluoxetine**
DB00514	Dextromethorphan
DB01173	**Orphenadrine**
DB01242	Clomipramine
DB01364	**Ephedrine**
DB04896	**Milnacipran**
DB06204	Tapentadol
DB06700	**Desvenlafaxine**
DB08918	**Levomilnacipran**

Figure S3. Illustration of the dendogram enrichment analysis for selecting repurposable drugs. The analysis is performed for the dataset of 101 compounds composed of 27 FDA-approved (known) and 74-PM predicted (potentially repurposable) drugs for hDAT. The compounds are clustered based on the correlation cosine of their latent vectors computed by PMF applied to DrugBank. Cluster enrichment is calculated as the ratio of *observed* fraction of known drugs to the *expected* fraction (0.365 in this case), a higher number indicating that the cluster is populated by a large number of known drugs. One of the clusters is distinguished by high enrichment (here colored *green*). This cluster is selected for further examination of the corresponding predicted drugs (listed on the *right* table) (see **Figure 2**). Same analysis is performed for clusters obtained with two additional distance metrics, Euclidian and Mahalanobis (*not shown*). Repurposable drugs commonly predicted to lie in highly enriched clusters, by at least two different metrics are listed as candidates. Eight such drugs are obtained (written in boldface), with orphenadrine ranking at the top of the list based on PMF analysis.

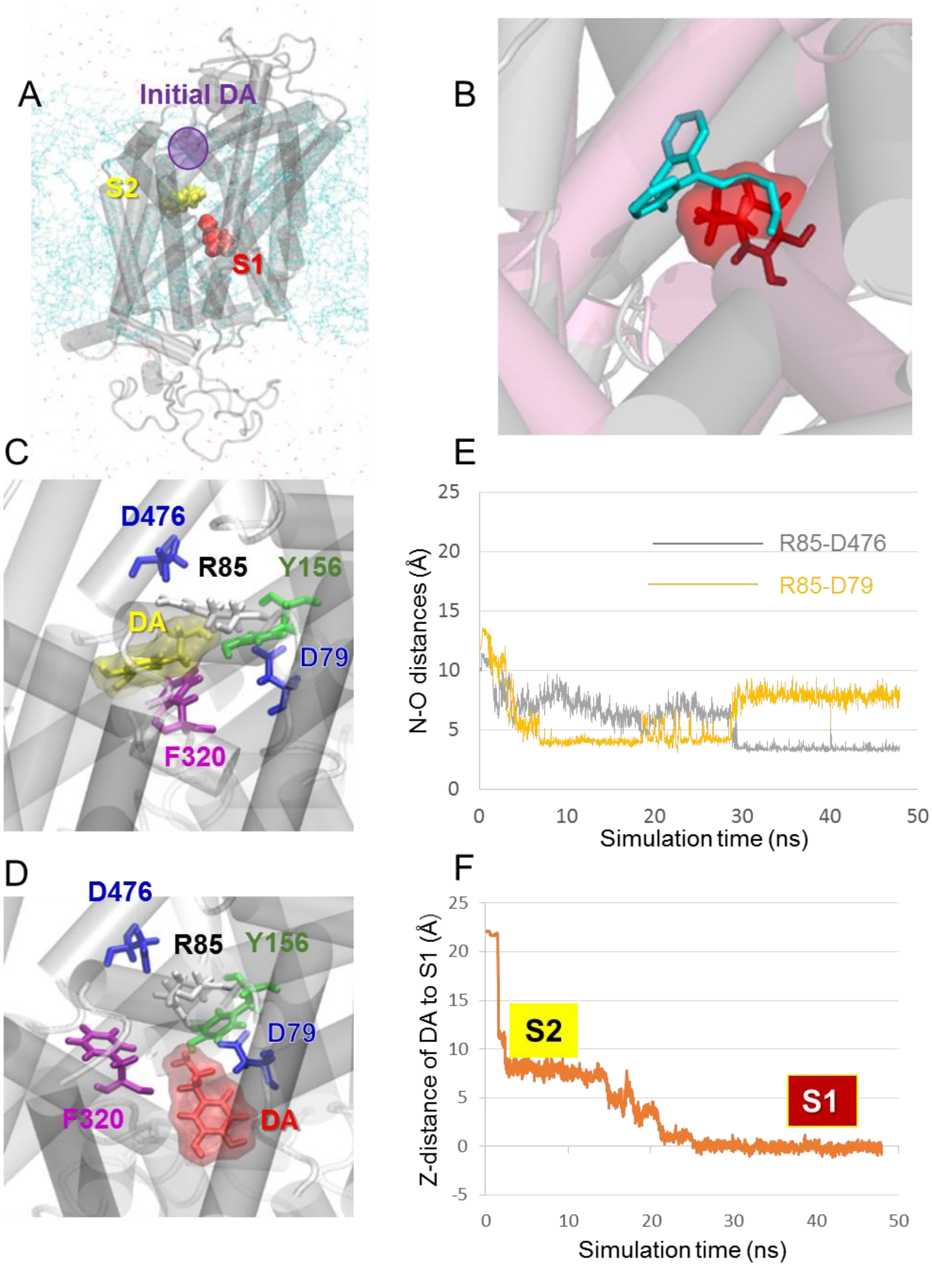

Figure S4. Binding of DA from the EC region to site S1. Initially, a DA molecule was placed ~20 Å away from site S1 near the entrance of the EC vestibule. (**A**) Sites S2 (*yellow*) and site S1 (*red*), where DA is observed to be temporarily stabilized prior to translocation through the transporter. **(B)** Alignment of DA- (*red*) bound hDAT (*silver*) with nortriptyline- (*cyan*) bound dDAT (*pink*) (PDB: 4M48). (**C**) and (**D**) Representative snapshots illustrating the binding of DA to site S2 (**C**) and S1 (**D**). Key coordinating residues include F320 (*purple*), R85 (*white*), Y156 (*green*), D79 and D476 (*blue*). Time evolution of (**E**) N-O distances for the pairs R85-D476 (*gray*) and R85-D79 (*orange*) and (**F**) Z-distance of DA from the S1 site. Note that R85 switches salt-bridge forming partner (from D79 to D476) as DA moves from S2 to S1.

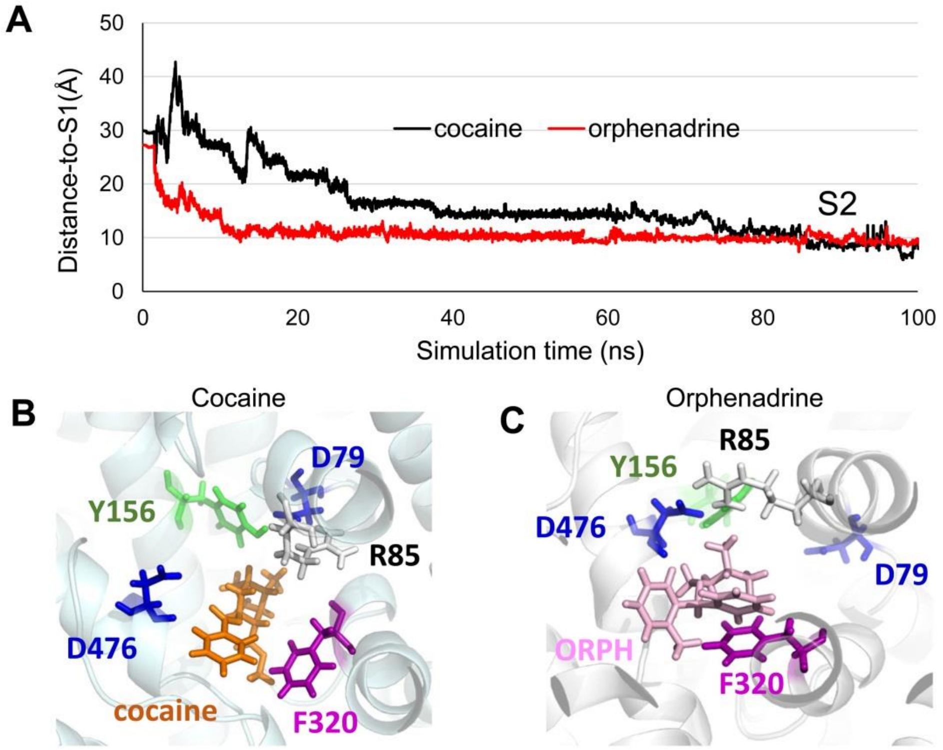

Figure S5. Cocaine and orphenadrine diffuse into the EC vestibule to bind near site S2 in the OF*o* state of hDAT. (**A**) Time evolutions of the instantaneous positions of cocaine (*black*) and ORPH (*red*), along the z-axis normal to membrane, with respect to primary site S1 (where *z* = 0). (**B**) Top view of cocaine (*orange*) binding geometry to site S2, along with the positions of the EC gating residues R85, D476, F320, and Y156 which are all open/dissociated. (**C**) Top view of orphenadrine (*pink*) binding to site S2. Diagrams are constructed using snapshots from the end of the two 100 ns runs.

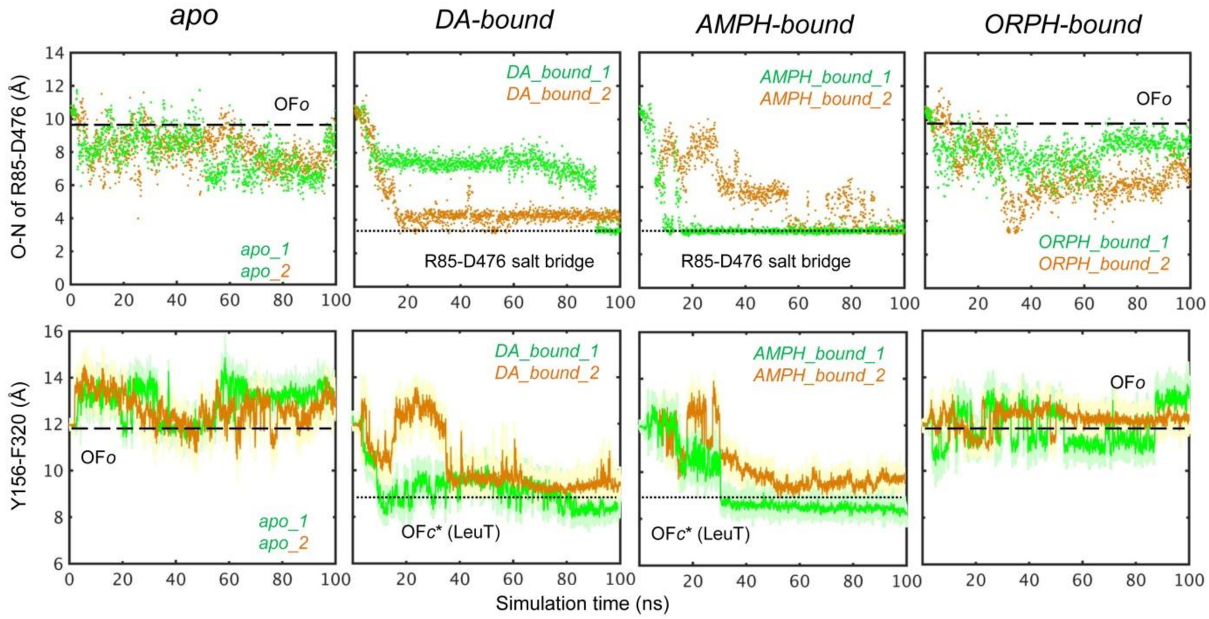

Figure S6. Time evolution of the distances between the two EC gating residue pairs. (***top panel***) O-N distances between salt-bridge forming residue pairs R85 and D476. Salt bridges serving as the outer EC gate were formed in the simulations of DA-bound and AMPH-bound hDAT, but not in the apo form. The ORPH-bound hDAT showed intermittent formation of salt bridges which were not stable enough to promote the closure of the EC vestibule. (***bottom panel***) Association between the aromatic residues Y156 and F320 that form the inner EC gate. EC gate closure took place in DA-bound and AMPH-bound systems, but not in the apo and ORPH-bound hDAT. Results were taken from two independent runs (colored *green* and *light brown*) for apo, DA-bound, AMPH-bound, and ORPH-bound hDAT simulations.

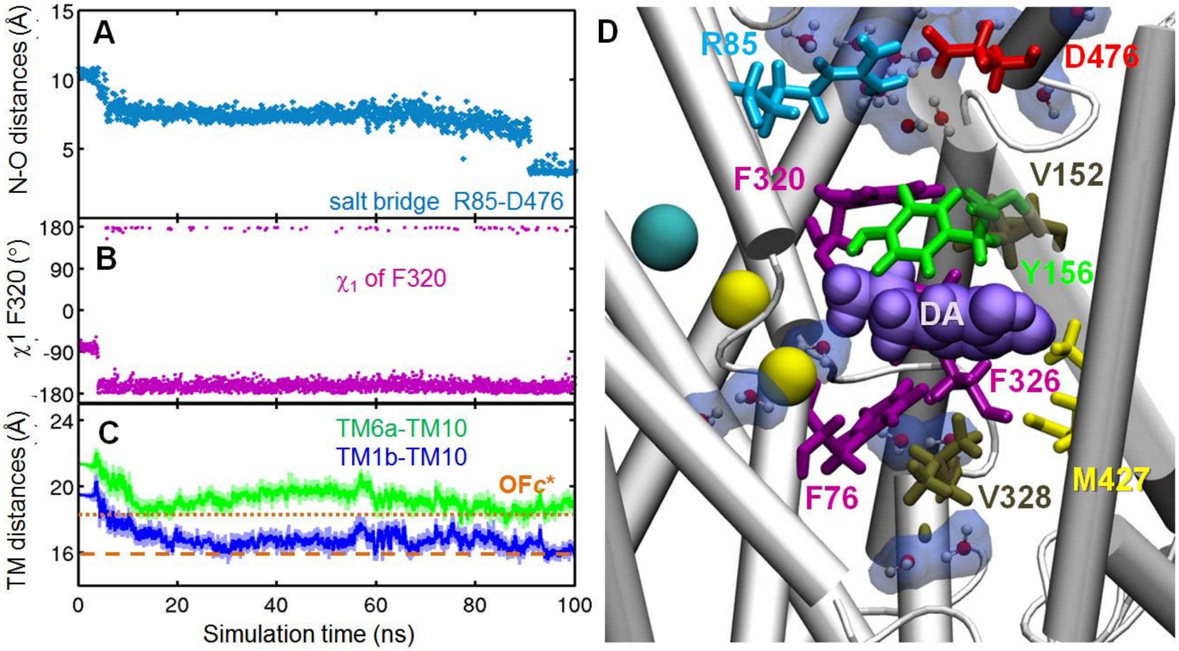

Figure S7. Binding of dopamine promotes the transition of hDAT into outward-facing closed (OFc*) state. Time evolutions of (**A**) N-O distance of R85-D476. Salt-bridge was formed around 90 ns; (**B**) χ_1 of F320. F320 flipped on top of dopamine as χ_1 changed from -80±15° to 170±15° around 5 ns; (**C**) CoM distances of EC-exposed TM segments TM6a-TM10 (*green*) and TM1b-TM10 (*blue*). (**D**) In the *OFc** state, side view of the binding site for dopamine (*purple* vDW) is highly dehydrated and occluded to both EC and IC region. Water molecules in the EC and IC vestibules are shown in CPK format with semi-transparent *cyan.* Results are taken from *DA-bound-1*.

References:

1. Wang J, Wang W, A. KP, Case DA. Automatic atom type and bond type perception in molecular mechanical calculations. *J Mol Graph and Modelling* (2006) **25**:247260.
2. Case DA, Cheatham TE, Darden TOM, Gohlke H, Luo RAY, Merz KM, et al. The Amber Biomolecular Simulation Programs. *J comput chem* (2005) **26**(16):1668-88.
3. Hoover W. Canonical dynamics: equilibrium phase-space distributions. *Phys Rev A* (1985) **31**:1695.
4. Nosé S. A Unified formulation of the constant-temperature molecular-dynamics methods. *J Chem Phys* (1984) **81**:511-9.
5. Stockner T, Montgomery TR, Kudlacek O, Weissensteiner R, Ecker GF, Freissmuth M, et al. Mutational analysis of the high-affinity zinc binding site validates a refined human dopamine transporter homology model. *PLoS Comput Biol* (2013) **9**(2):e1002909.
